# Recumbency as an Equine Welfare Indicator in Geriatric Horses and Horses with Chronic Orthopaedic Disease

**DOI:** 10.3390/ani11113189

**Published:** 2021-11-08

**Authors:** Zsofia Kelemen, Herwig Grimm, Mariessa Long, Ulrike Auer, Florien Jenner

**Affiliations:** 1 Equine Surgery Unit, University Equine Hospital, Department of Companion Animals and Horses, University of Veterinary Medicine Vienna, Veterinaerplatz 1, 1210 Vienna, Austria; Zsofia.Kelemen@vetmeduni.ac.at; 2 Unit of Ethics and Human-Animal-Studies, Messerli Research Institute, University of Veterinary Medicine Vienna, Medical University of Vienna, University of Vienna, Veterinaerplatz 1, 1210 Vienna, Austria; Herwig.Grimm@vetmeduni.ac.at (H.G.); Mariessa.Long@vetmeduni.ac.at (M.L.); 3 Anaesthesiology and Perioperative Intensive Care Medicine Unit, Department of Companion Animals and Horses, University of Veterinary Medicine Vienna, Veterinaerplatz 1, 1210 Vienna, Austria

**Keywords:** welfare, horse, equine, sleep, lying, time budget, locomotion, geriatric, orthopedic, recumbency

## Abstract

**Simple Summary:**

Horses have to lie down to achieve rapid eye movement (REM) sleep. Horses that do not lie down for environmental reasons or pain suffer from an REM sleep deficiency that negatively affects their welfare and health. The present study aimed to assess the influence of chronic orthopedic disease and old age on the time horses lie down. Wearable automated sensor technology was used to monitor the time 83 old and young adult horses with or without chronic lameness spent lying down, moving, or standing. Interestingly, neither age nor lameness due to chronic orthopedic disease significantly influenced the time spent lying down. Horses showing symptoms of REM sleep deficiency had shorter lying times and reduced times spent moving, indicating a general compromise of their well-being. The study shows that wearable sensor technology can be used to identify horses with short recumbency times at risk for REM sleep deficiency. Furthermore, the technology can be used to assess and monitor equine welfare objectively and optimize husbandry conditions so that old horses and horses suffering from chronic orthopedic conditions can achieve lying-down times comparable to younger, healthy horses.

**Abstract:**

Recumbency is a prerequisite for horses achieving rapid eye movement (REM) sleep and completing a full sleep cycle. An inability to lie down due to environmental insecurities or pain results in REM sleep deficiency, which can cause substantial impairment of welfare and health. Therefore, the present study used wearable automated sensor technology on 83 horses housed in an animal sanctuary to measure and compare the recumbency, locomotion, and standing time budgets of geriatric horses with and without chronic lameness to younger adult sound and lame horses. Recumbency times ranged from 0 to 319 min per day with an overall mean of 67.4 (±61.9) minutes; the time budget for locomotion was 19.1% (±11.2% s.d.) and for standing 75.6% (±13.1 s.d.). Interestingly, neither age nor lameness due to chronic orthopedic disease had a significant influence on recumbency times in this study. Eight horses showed symptoms of REM deficit. These horses had significantly shorter lying times (7.99 ± 11.4 min) and smaller locomotion time budgets than the other horses enrolled in this study (73.8 ± 61.8 min), indicating a general compromise of well-being. Thus, wearable sensor technology can be used to identify horses with low recumbency times at risk for REM sleep deficiency and to assess and monitor equine welfare objectively.

## 1. Introduction

Recumbency is a prerequisite for horses achieving rapid eye movement (REM) sleep and complete a full sleep cycle [1,2,3,4]. While adult horses sleep only 2.5–5 h/day, 80% of which is in a standing position, they need a minimum of 30 min of recumbency per day to achieve 3.5–4.5 min of REM sleep and avoid REM sleep deprivation with excessive secondary drowsiness and collapse [1,2,3,4,5,6,7,8,9,10,11,12,13,14,15,16]. However, as a prey species, horses only lie down when they feel comfortable to do so [2,4,5,14,17,18,19,20,21,22,23]. Hence, measuring lying behavior is an essential component of equine welfare assessment [12,13].

Adult (>4 years) horses, both domestic and (semi-)feral, spend 3–15% (43–216 min) of their total daily time budget (=percentage of time spent on specific activities) in recumbency, of which about 20 min or 15% are spent in lateral and the rest in sternal recumbency [1,7,12,16,20,21,24,25,26,27,28,29,30,31,32,33]. The consistency of lying times between the vastly different domestic and free-ranging living conditions emphasizes the importance of recumbency as basic maintenance behavior. In comparison, the time budgets for eating, resting standing, and locomotion vary greatly between adult domestic and free-ranging horses, with the latter spending 50.82–66.6% foraging, 12.9–29.3% of their day standing, and 4.3–13.4% in movement (excl. grazing), while domestic horses divide their time between 10–64% eating, 15.6–68% standing, and 2.5–19.3% locomotion. 

Sleep is a basic maintenance behavior, essential for physiological and cognitive function. Horses sleep in a polyphasic pattern, distributed over 5–7 episodes, with most sleep occurring between midnight and 4:00 am [2,3,4,5,8,10,12,14,32,33,34,35,36,37]. Based on postural and behavioral indicators and specific cortical electronic activity, four sleep–wakefulness states are differentiated: wakefulness (18 h/d [3]), drowsiness (2 h/d [3]), slow-wave sleep (SWS, 3 h/d [3]), and paradoxical or rapid eye movement (REM) sleep (<1 h/d [3]) with only the latter two counting toward the total sleep time budget of typically 2.9–3.5 h/d (10–21% of the total daily time budget) [3,5,6,7,8,12,16,31,32,33,38,39,40]. REM sleep represents the smallest proportion (10–15%) of the total sleep time, while SWS, at 65% takes up most sleep time [4,5,7]. While sleep in horses, in contrast to most other species, is not uniquely associated with recumbency, as horses can go through SWS in both standing and recumbent positions, the muscle atonia associated with REM sleep requires sternal or lateral recumbency [1,2,3,4,5,7,11,14,16,31]. Indeed, since horses usually fall asleep shortly after lying down, recumbency can be used as an inferred measure of sleep [7,11,12,16]. Polysomnographic studies demonstrated that horses were in REM sleep 29.7% of the time spent sleeping in sternal and 33.7% of the time in lateral recumbency, with SWS accounting for the remaining time [16,39]. As a reduction in recumbent sleep states cannot be compensated for by increased sleep time standing, the reluctance of a horse to enter a recumbent position causes REM sleep deficiency and can have substantial effects on health and quality of life [14]. 

The duration of lying, and with it the quality and length of sleep, is affected by various environmental influences, including the availability of a suitable lying area, space allowance, the presence and type of bedding, and lighting conditions [11,14,19,20,22,23,36,39,41,42,43,44,45,46,47]. Also, age influences the lying times with foals (up to 53.1% in domestic foals [10] and up to 15% in semi-feral foals [48]) and young horses (<2 years; up to 27% in domestic horses raised for meat production [49,50]; up to 8% in semi-feral horses [27]), who spend more time in recumbency than adults (3–15% in domestic and semi-feral horses [1,7,12,16,20,21,24,25,26,27,28,29,30,31,32,33,51,52]); however, the influence of old age on lying times has not yet been reported. In addition, painful conditions can modify lying times [4,18,53,54,55,56,57]. While recumbency is increased in acute pain due to colic or acute laminitis, it has recently been reported to decrease in horses suffering from angular limb deformities; analgesia administration resulted in a return to regular lying times [4,18,53,54,55,56,57]. However, the effect of other chronic orthopedic diseases such as osteoarthritis, tendinopathy, or chronic laminitis on recumbency has not been evaluated yet. The paucity of data on equine recumbency times is mainly due to the time and resource requirements for measuring this predominantly nocturnal behavior by direct or video observation without affecting the behavior studied [58,59]. The individual variation of the equine behavioral circadian rhythm requires detailed surveillance over several successive days [12,60,61,62,63]. However, to date, only a few studies, four in (semi-)feral and five in domesticated horses, measured recumbency times for a minimum of 24 continuous hours [12,24,25,26,27,28,29,48,49,50]. Recent advances in biotelemetry, and biologging, using wearable automated tracking equipment, provide increased objectivity and new opportunities to remotely quantify behavior at scales and temporal resolutions that were not previously possible [12,64]. These new technologies facilitate accurate time budget analysis over several successive days as an objective, quantitative measure of behavior, and have the potential to become a reliable tool for on-farm assessment of equine welfare. 

Given the aging equine population and the prevalence of musculoskeletal problems in horses [65,66,67,68,69,70,71,72,73,74], the present study aimed to assess the influence of chronic musculoskeletal disease and old age on the lying time budgets of horses using wearable sensor technology. We hypothesize that geriatric horses and horses suffering from chronic orthopedic discomfort spend less time recumbent than healthy adult control horses. 

## 2. Materials and Methods

### 2.1. Horses, Housing and Management Conditions

This prospective, observational cohort study was carried out in 83 horses, 39 warmbloods, 17 draft horses, and 27 horses of other breeds (Supplementary Table S1), owned by an animal sanctuary. Horses were housed in familiar environments, in individual box stalls (16 m^2^, *n*= 55) or group housing (2–10 horses/group, ≥11 m^2^/horse, *n* = 27), and had daily paddock or pasture turn-out (season and weather-dependent) in groups that had been stable for at least 6 months. Lying surfaces were bedded with straw (*n* = 64) or shavings (*n* = 18). Horses had ad libitum access to water and were fed a predominantly grass and hay-based diet ad libitum or rationed, depending on their nutritional requirements. 

**Figure 1:**
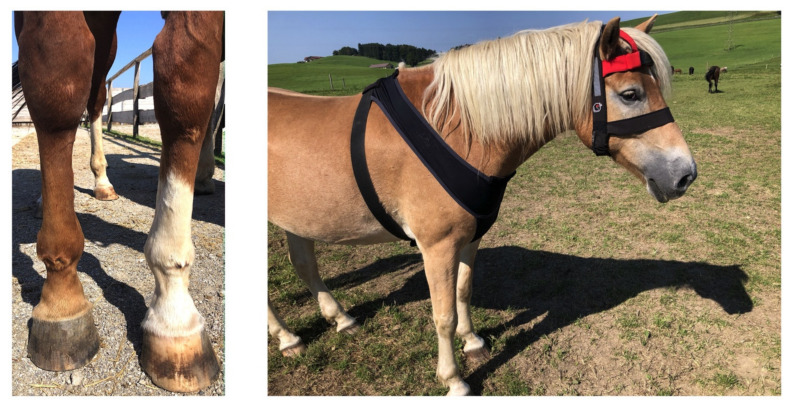
The photograph on the left shows the pathognomonic skin lesions over the dorsal aspect of both metacarpophalangeal (fetlock) joints and carpi of a horse with chronic REM deficit. The photograph on the right displays a horse equipped with the automated equine monitoring system (Trackener^®^) in the lycra bib.

Prior to inclusion in the study and every three months for the duration of the study, horses were examined by the same veterinarian, and their physical health and body condition score (BCS, range 1 (=extremely emaciated) to 9 (=extremely fat) [75]) were recorded. Based on their age, and physical and orthopedic exams, horses were assigned to one of four health/age groups: (1) horses younger than 20 years with chronic orthopedic diseases (chronic lameness > 1 (on the American Association of Equine Practitioner (AAEP) scale), *n* = 31); (2) geriatric horses (≥20 years) with chronic orthopedic disease (*n* = 40); (3) sound (lameness ≤ 1) geriatric horses (*n* = 7); and (4) sound horses younger than 20 years (control group, *n* = 5). Horses with cardiovascular, respiratory, or abdominal disease or acute onset or exacerbation of lameness were excluded from the study. Horses that were observed to collapse or that exhibited the associated pathognomonic skin lesions on the dorsal aspect of their carpi and fore fetlocks (Figure 1, Supplementary Videos S1 and S2) were considered REM-sleep deprived. Horses that were included in the study but developed additional health problems or an acute exacerbation of their musculoskeletal disease after the first tracking round was completed did not participate in additional tracking rounds to avoid masking the effects of the chronic conditions that were the focus of this study with acute disease. 

### 2.2. Automated Equine Monitoring

Using the Trackener^®^ (London, UK) automated equine monitoring system [76], horses were tracked 1–3 times within 15 months for a minimum of 60 continuous hours (max of 360 h), each with horses that showed abnormal recumbency patterns being tracked longer and repeatedly. The Trackener^®^ system measures the horse’s body position (standing, sternal, or lateral recumbency), gait, speed, rein (left, straight, right), and location [76]. The wearable horse unit (140 × 50 × 30 mm, weight: 190 g), containing a MEMS 3-axis accelerometer, a gyroscope, a barometer, a temperature sensor, and a GPS, is carried within a special lycra bib (Figure 1).

The wearable horse units send the data via GSM communication (3G network) to the cloud. An artificial intelligence algorithm analyses the data and displays the amount of time the horse spent resting (detailed by body position into standing, sternal, or lateral recumbency) and active (divided into walk, trot, and canter). The activity data measured by the Trackener^®^ device have been previously validated in an equine hospital setting against direct observation of horses’ activities recorded manually based on CCTV recordings, which yielded a mean agreement of 95.7% [76]. 

Time budgets are presented as minutes per hour and day in the corresponding Trackener^®^ app for each behavior category. In addition, data differentiated between recumbency versus upright body position and between standing and movement are provided in csv format for further analysis. Recorded lying times of less than 1 min were considered artifacts and not included in the recumbency time budget. To facilitate reading, recumbency times are indicated in minutes rather than the percentage of the 24 h time budget used for movement and standing, as the small lying time budgets expressed in percentages were too cumbersome to interpret in the context of horses’ daily behavioral routines. 

### 2.3. Statistical Analysis

Continuous variables were expressed as mean ± standard deviation (s.d.), and categorical variables were expressed as percentages. The effect of time and horse on time budgets was analyzed using a Brown–Forsythe ANOVA to account for the difference in group size. A generalized linear model using time budgets as target variables and age, lameness (yes/no), REM sleep deficiency (yes/no), BCS, sex (gelding/mare), breed group (warmblood, draft, or other), and pasture (yes/no) as explanatory variables was calculated. Statistical analyses were carried out using Graphpad Prism version 9 (Graphpad Software, San Diego, CA, USA) [77]. A *p*-value < 0.05 was considered significant.

### 2.4. Ethics Statement

This study was non-invasive and entailed only monitoring the horses under their current conditions of life. No specific veterinary treatments or interventions were carried out for the purpose of this study. The study was thus reviewed by the Institutional Ethics Committee of the University of Veterinary Medicine Vienna (ETK-152/09/2019) in accordance with the “Good Scientific Practice. Ethics in Science and Research” guidelines implemented at the University of Veterinary Medicine, Vienna and national legislation, and ethical approval was waived.

## 3. Results

### 3.1. Horses and Tracking 

Of the 83 horses included in this study, 38 were mares and 45 geldings. Their age ranged from 2 to 32 years (20.7 ± 6.2 years), and their body condition score from 3 to 7 (5.7 ± 1.1) (Supplementary Table S1). In health/age group 1 (lame, <20 y, *n* = 31), horses’ mean age was 15.7 (±4.2 s.d.); in group 2 (lame, ≥20 y, *n* = 40), 24.9 (±3.5 s.d.); in group 3 (sound, ≥20 y, *n* = 7), 25 (±3.5 s.d.); and in group 4 (sound, <20 y, *n* = 5), 12 (±5.8 s.d.). Eight horses (age: 24.5 ± 5.5), all of whom suffered from chronic orthopedic disease) showed signs of REM sleep deficit (collapse or pathognomonic skin lesions observed). Four of the eight REM-sleep-deprived horses had single stalls with straw bedding, 2 had single stalls with shavings, and 2 were in small group housing with straw bedding. 

BCS was significantly affected by breed (*p* = 0.0048, F(Dfn, DFd) = 5.598 (2, 115)) and chronic lameness (*p* = 0.002, F(Dfn, DFd) = 9.974 (1, 115)), but not by age (*p* = 0.4029), REM sleep deficiency (*p* = 0.0686), sex (*p* = 0.1784), pasture access (*p* = 0.6774), bedding (*p* = 0.5547), or housing (*p* = 0.1634) conditions.

All horses tolerated the wearable horse unit well, and no dermal irritations were observed. Data collection and transfer functioned well in 131 tracking cycles, and no technical problems were encountered.

### 3.2. Time Budgets for Lying and the Influence of Age, Lameness, Presence of REM Deficit Symptoms, Sex, BCS, Breed, and Pasture Access on Recumbency

**Figure 2:**
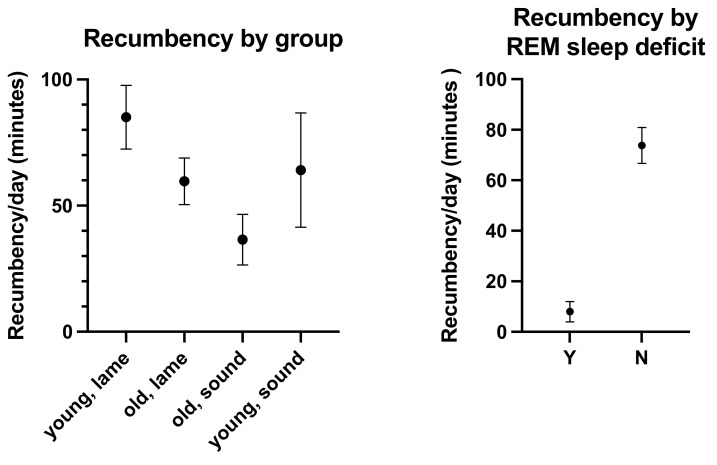
Comparative recumbency times (mean +/−SEM minutes per day) of the horses enrolled in this study by age/lameness group and by the diagnosis of REM deficit (Yes (Y) and No (N)).

The overall mean duration of recumbency was 67.4 min (±61.9 s.d. range: 0–319 min) per day. Young, lame horses were recumbent for 85 min (±70.3 s.d.); old, lame horses for 59.7 min (±58.5 s.d.); old, sound horses for 36.5 min (±26.5 s.d.); and young, sound horses for 64.1 min ((±50.7 s.d.) (Figure 2, Table 1, Supplementary Tables S1 and S2). The effects of age (*p* = 0.7408, F(Dfn, DFd) = 0.11 (1, 115)) and lameness (*p* = 0.3072, F(Dfn, DFd) = 1.052 (1, 115)) were not statistically significant. 

Horses with clinically established REM sleep deficit had significantly (*p* = 0.0003, F(Dfn, DFd) = 14.25 (1, 115)) shorter lying times (7.99 min ± 11.4 s.d.) than other horses (73.8 min ± 61.8 s.d., Table 1, Figure 2). Also, breed had a statistically significant effect on recumbency times (*p* = 0.0052, F(Dfn, DFd) = 5.513 (2, 115)), with draft horses (88 ± 84 min/d) lying significantly more than warmbloods (58 ± 59 min/d) or horses of other breeds (69 ± 67 min/d). Furthermore, BCS (*p* = 0.0263, F(Dfn, DFd) = 2.867 (4, 115)) significantly influenced lying times. Horses with a BCS of 4 had longer recumbency times (122 ± 122 min/d) than horses with lower (BCS 3: 60 ± 55 min/d ) or higher BCS (BCS 5: 50 ± 47, BCS 6: 73 ± 63, BCS 7: 51 ± 52). However, bedding (straw versus shavings, *p* = 0.9427, F(Dfn, DFd) = 0.0052 (1, 115)), housing (single box stall versus group housing, *p* = 0.735, F(DFn, DFd) = 0.1151 (1, 115)), sex (*p* = 0.3094, F(Dfn, DFd) = 1.042 (1, 115)), and pasture access (*p* = 0.2901, F(Dfn, DFd) = 1.13 (1, 115)) did not significantly affect lying times. 

**Figure 3:**
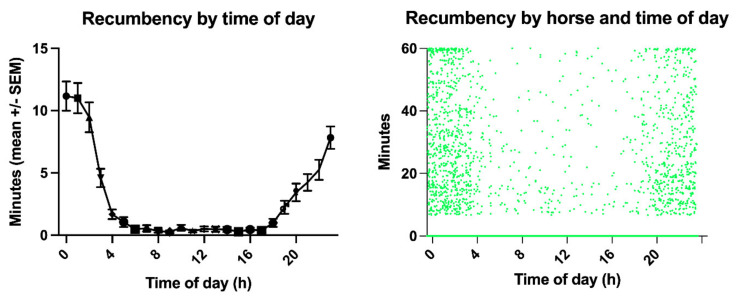
The left graph illustrates the recumbency times throughout the day, showing the primarily nocturnal recumbency distribution with a peak between midnight and 4:00 a.m. The right graph, the scatter plot of the recumbency minutes per horse, demonstrates the individual variation of the circadian sleep rhythm of the horses enrolled in this study.

Time of day and horse had a significant influence on lying time budgets (*p* < 0.0001, Figure 3, Supplementary Figure S1). Eight horses slept only at night (0:00–4:00), one only during the day (4:00–20:00), the others distributed over the 24 h day. Between 0:00 and 4:00, lying bouts lasted 2 to 57 min (16 ± 15 s.d.); in the time between 4:00–20:00, 7–12 min (10 ± 7 s.d.); and between 20:00–0:00, 7–16 min (10 ± 12 s.d.). 

**Figure 4:**
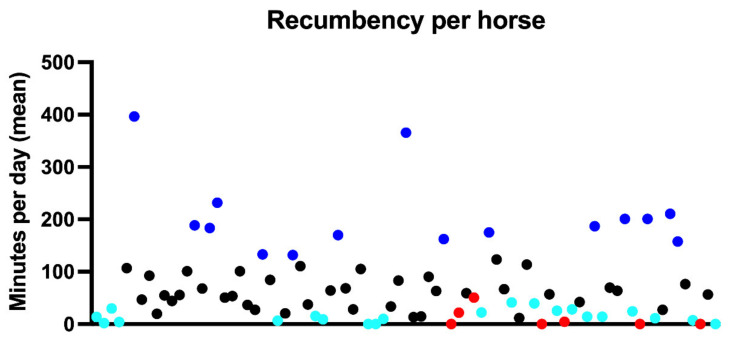
Recumbency time budget (mean %) per horse (horses are distributed alphabetically along the x-axis). Horses with established REM deficit are highlighted in red, turquoise indicates horses with less than 30 min sleep per day throughout the study period, and horses that lay down more than average are marked in blue.

Twenty-nine horses were recumbent for less than 30 min per day throughout the entire study period, 13 of which all suffered from chronic orthopedic disease and did not lie down at all for more than 72 h (Figure 4); five of these horses lay down during subsequent tracking periods for 2–48 min. Thirty horses were consistently recumbent for more than one hour per day (Figure 4).

**Figure 5:**
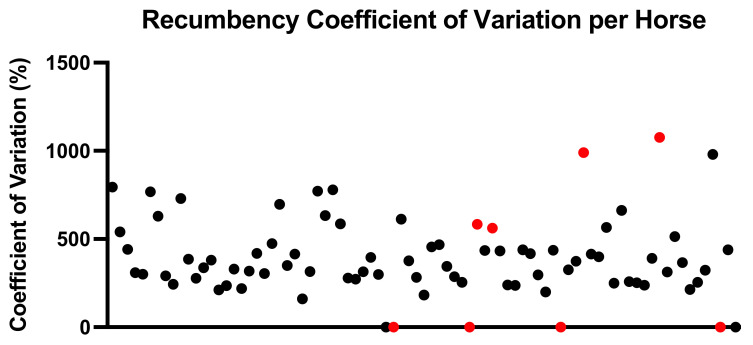
Coefficient of Variation (%) of recumbency times. Horses (distributed alphabetically along the x-axis) with established REM deficiency symptoms are indicated in red.

The coefficient of variation was, on average, 370% (range 0–1076%) for evidently REM-deficient horses and 396% (range 0–990%) for other horses (Figure 5). Horses with REM deficit symptoms notably either had a coefficient of variation of 0% because they did not lie down at all, or above 500% because they lay down only rarely. In total, 19 horses had a coefficient of variation above 500%, and 6 of 0%, all of whom had recumbency times well below the population average. 

### 3.3. Time Budgets for Locomotion and the Influence of Age, Lameness, Presence of REM Deficit Symptoms, Sex, BCS, Breed, and Pasture Access on Movement

**Figure 6:**
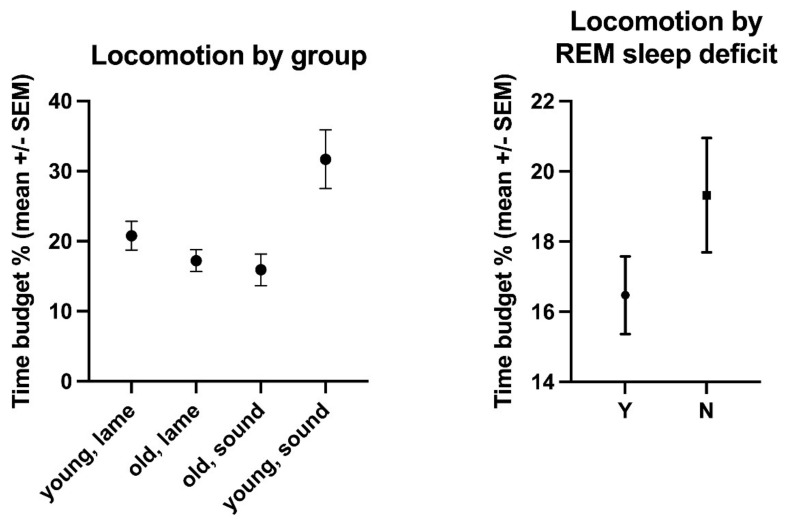
Comparative locomotion time budgets (mean % of 24 h +/− SEM) of the horses enrolled in this study by age/lameness group and by the diagnosis of REM deficit (Yes (Y) and No (N)).

**Figure 7:**
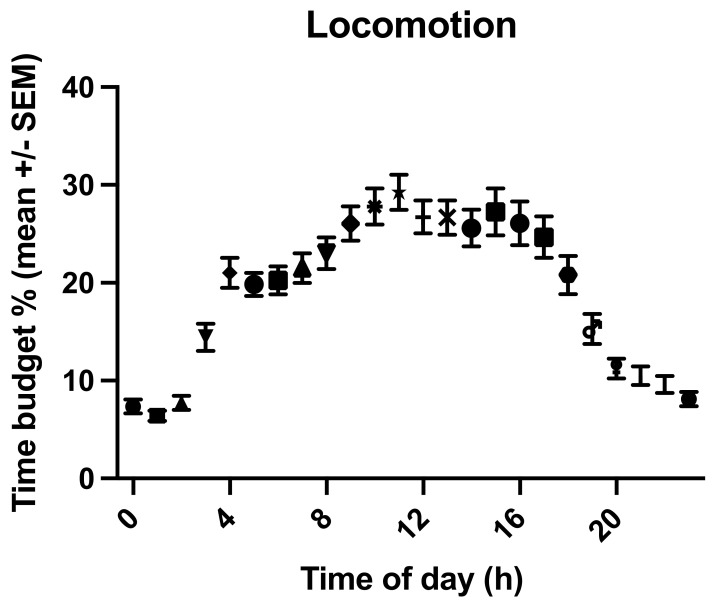
Graph of the locomotion time budget (mean % of 24 h +/−SEM) throughout the day, showing the movement peak during daytime hours and less movement at night.

The overall mean time budget for locomotion was 19.1% (±11.2% s.d., range: 4.09–55.8%, Supplementary Tables S1 and S3). Young, lame horses moved for 20.79% (±15.13% s.d.) of their day, old, lame horses for 17.23% (±12.05% s.d.), old, sound horses for 15.92% (±6.8% s.d.), and young, sound horses for 31.7% (±11.85% s.d.) (Figure 6, Supplementary Tables S1 and S3). The effect of age (*p* = 0.0302, F(Dfn, DFd) = 4.82 (1, 114)) but not of lameness (*p* = 0.6867, F(Dfn, DFd) = 0.1635 (1, 114)) on movement time budgets was statistically significant, with young horses moving more (22.39% ± 15.15%) than old horses (17.05% ± 11.46%). Horses with evident REM deficits moved significantly less (16.5% ± 5.42%) than other horses (19.3% ± 7.07%, *p*= 0.0383, F(Dfn, DFd) = 4.392 (1, 114)). Unsurprisingly, horses with access to pasture moved significantly more (33.31% ± 12.74% of their time budget) than horses with more restricted turn-out (12.56% ± 7–08%, *p* < 0.0001, F(Dfn, DFd) = 119.7 (1, 114)). Bedding (*p* = 0.5511, F(Dfn, DFd) = 0.3575 (1, 114)), housing (*p* = 0.066, F(Dfn, DFd) = 3.446 (1, 114)), sex (*p* = 0.5827, F(Dfn, DFd) = 0.3036 (1,114)), breed (*p* = 0.1492, F(Dfn, DFd) = 1.935 (2, 114)), and BCS (*p* = 0.8084, F(Dfn, DFd) = 0.3999 (4, 114)) had no significant effect on movement. Time of day had a significant influence on the time budgets for locomotion (*p* < 0.0001, Figure 7), but horse did not (*p* = 0.7124). 

### 3.4. Time Budget for Standing and the Influence of Age, Lameness, Presence of REM Deficit Symptoms, Sex, BCS, Breed, and Pasture Access on Standing Times

**Figure 8:**
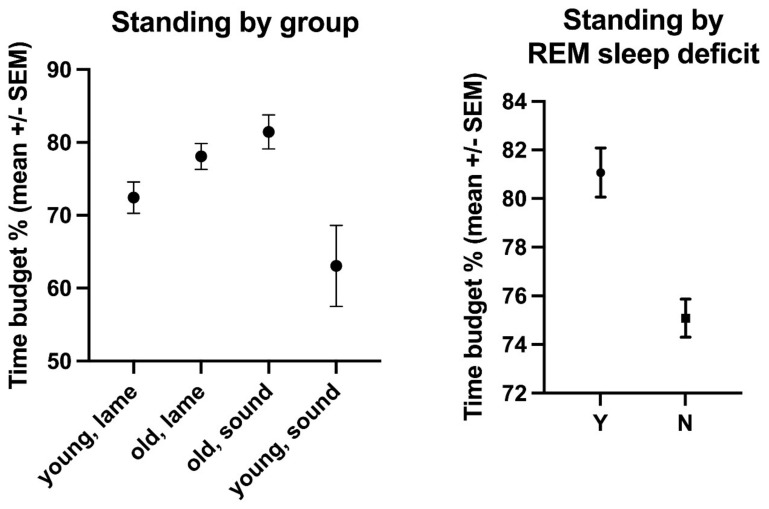
Comparative standing time budgets (mean % of 24 h +/−SEM) of the horses enrolled in this study by age/lameness group and by the diagnosis of REM deficit (Yes (Y) and No (N)).

The overall mean time budget for standing was 75.6% (±13.1 s.d., range: 32.2–95.9%, Supplementary Tables S1 and S4). Young, lame horses were standing for 72.43% (±15.99% s.d.) of their day, old, lame horses for 78.08% (±13.74% s.d.), old, sound horses for 81.45% (±7.03% s.d.), and young, sound horses for 63.05% (±15.7% s.d.) (Figure 8, Supplementary Tables S1 and S2). The effect of age (*p* = 0.0489, F(Dfn, DFd) = 3.965 (1, 114)) but not lameness (*p* = 0.7067, F(Dfn, DFd) = 0.1424 (1, 114)) was statistically significant, with old horses standing more (78.53 ± 13.06) than young horses (71.1 ± 16.23). REM sleep deficit (*p* = 0.6012, F(Dfn, DFd) = 0.2747 (1, 114)), BCS (*p* = 0.9484, F(Dfn, DFd) = 0.1799 (4, 114)), bedding (*p* = 0.5069, F(Dfn, DFd) = 0.4433 (1, 114)), housing (*p* = 0.126, F(Dfn, DFd) = 2.376 (1, 114)), sex (*p* = 0.4451, F(Dfn, DFd) = 0.5872 (1, 114)), and breed (*p* = 0.9769, F(Dfn, DFd) = 0.0234 (2, 114)) had no statistically significant effect on standing times, but pasture access (*p* < 0.0001, F(Dfn, DFd) = 96.36 (1, 114)) did. Horses on pasture stood significantly less (60.4% ± 14.13% of their time budget) than those with more restricted turn-out (82.38% ± 8.77%).

**Figure 9:**
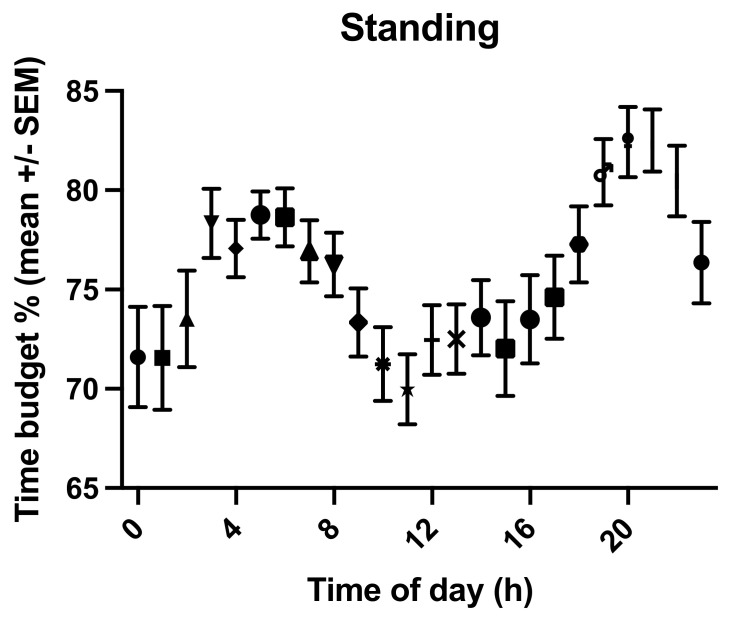
Graph of the standing time budget (mean % of 24 h +/−SEM) throughout the day with a peak in standing times in the morning and evening.

Time of day significantly influenced standing time budgets (*p* < 0.0001, Figure 9), but horse did not *p* = 0.4778).

## 4. Discussion

Recumbency times of the primarily geriatric and lame population of horses in this study ranged from 0 to 319 min per day, with a mean of 67.4 min. Interestingly, neither age nor lameness due to chronic orthopedic disease significantly influenced recumbency times in this study. Young, lame horses lay down for 85, old, lame horses for 59.7, old, sound horses for 36.5, and young, sound horses for 64.1 min per day. As sleep duration is inversely proportional to the risk of predation, horses sleep only for short periods, typically for 2–15 min, and rarely remain recumbent for longer than 30 min at a time [1,7,12,16,20,21,24,25,26,27,28,29,30,31,32,33]. The occurrence and duration of recumbency depend on the horse’s ability to find a comfortable and safe place to lie [3,4,5,47]. Correspondingly, decreased lying time budgets are associated with unsuitable environmental conditions, stress, social insecurity, and pain [18,24,51,56,78]. Adaptions to a horse’s environmental conditions should therefore be considered if a horse shows insufficient lying times. 

Intriguingly, while (semi-)feral horses were observed to prefer open spaces for recumbency [2,7,11,27,28,34], in domesticated horses, recumbency times are longer in box stalls than in free-stall housing or on pasture [16,19,23]. Also, the effect of an individual’s hierarchical status on recumbency times depends on space availability [7,11,20,27,28,79,80]. In free-ranging horses, social rank has no effect on lying times, since the lack of spatial limitations under natural conditions seems to allow each individual within a group to satisfy their demand for recumbency [7,11,20,27,28,79,80]. In contrast, in group-housed domesticated horses, for whom a suitable lying area represents a potentially limited resource, larger lying surfaces increase the duration of recumbency and decrease the proportion of forcedly terminated lying bouts in low-ranking horses [7,11,20,27,28,79,80]. In the current study, neither pasture access nor housing conditions (single box stable versus group housing) significantly affected recumbency times, which may be due to the stable group composition and availability of adequate lying surfaces in the equine sanctuary. The effect of hierarchy on equine recumbency times was not assessed in this study, but further studies looking at the effect of social rank on recumbency times of group-housed horses are needed to establish evidence-based husbandry recommendations to improve equine welfare. 

Both (semi-)feral and domestic horses prefer dry, clean, and soft lying surfaces [11,14,19,20,21,22,23,31,36,39,41,42,43,44,45,46,47,80]. While straw bedding has been reported to increase recumbency times compared to shavings or rubber mats [19,20,21,22,41,42,43,44,45,47], there was no difference in lying times between horses with straw versus shavings bedding in this study. 

Visual and auditory stimuli have also been observed to influence equine recumbency and sleep times, with absent stimuli increasing SWS, but also especially REM sleep [2,3,5,14,32,38,39,41]. Unsurprisingly, artificial light overnight or during late-night checks, which may affect melatonin cycles and subsequently sleep patterns, decreases recumbency [14,38,39]. In contrast, music reduced alertness and increased recumbency periods, possibly by masking the occurrence of trivial novel environmental auditory stimuli [38,81,82]. 

In addition to environmental factors, personal factors also influence sleep. While age and sex had no impact on recumbency duration in this study, BCS and breed did significantly affect lying times, with draft horses (88 ± 84 min/d) lying down more than warmbloods (58 ± 59 min/d) or other breeds (69 ± 67 min/d). Surprisingly, moderately thin (BCS 4) horses had significantly longer recumbency times than horses in a thin (BCS 3) or moderate (BCS 5) to fleshy (BCS 7) condition. While in humans, sleep deprivation is associated with weight gain [83], the BCS of REM-sleep-deprived horses (5.38 ± 0.96) was similar to the other horses (5.71 ± 1.06). The BCS in this study (5.7 ± 1.1) was slightly above the midpoint of the scale, which is consistent with results obtained from other equine populations [84] and the emergence of equine obesity as one of the most important equine welfare issues in the western world [85,86]. However, the known association between breed type and BCS, with draft horses having the highest BCS [87], requires caution when interpreting results, and further studies, with selected breeds and a wider spread of BCS, to independently assess the influence of these variables on recumbency times. 

In this study, 8 out of 83 horses showed symptoms of REM deficit. These horses had significantly shorter lying times (7.99 min ± 11.4 s.d.) and locomotion time budgets (16.5% ± 5.42%) than the other horses enrolled in this study (73.8 min ± 61.8 s.d. lying, 19.3% ± 7.07% locomotion). To identify potential REM-deficient horses, it proved essential to track them for more extended periods, to determine whether they do not lie down at all, or only do so when exhausted or under specific environmental conditions. For example, one horse with physical problems lying down due to severe osteoarthritis in both carpi did lie down on an incline in the pasture, which made it easier for the horse to get up. 

REM-sleep deficiency due to recumbent sleep deprivation caused by illness, ethological deficits, or husbandry shortcomings typically manifests in excessive drowsiness and horses’ literally falling asleep while standing and partially collapsing before suddenly waking again, resulting in pathognomonic skin lesions on the dorsal aspect of the carpi and front fetlocks (Figure 1) [11]. The collapse is commonly incorrectly diagnosed as narcolepsy, a rare neurological sleep disorder characterized by daytime sleepiness, cataplexy, and sleep paralysis [9]. Recumbent sleep deprivation may have psychological or physical causes. Environmental-insecurity-associated excessive drowsiness arises when horses are not feeling comfortable to lie down due to social insecurity or fear of predation [2,3,4,5,7,9,10,14,15,17]. Also, stereotypical behaviors are associated with both suboptimal environmental conditions and decreased REM sleep [56,88,89,90,91,92]. Horses suffering from chronic musculoskeletal disease may be hesitant to assume recumbency due to pain or mechanical difficulties during lying down or rising. In contrast, administering analgesics to horses suffering from orthopedic pain increased lying times [12,18,51,56]. As REM-sleep deprivation is associated with hyperalgesia and persistence of chronic pain in other species [12,18,93,94], reduced recumbent sleep due to chronic pain may intensify pain sensation, further contributing to the problem [18]. REM sleep deficiency thus impairs welfare and health, necessitating further studies to investigate methods for early diagnosis and management as an essential basis to adjust husbandry and welfare decisions accordingly.

Interestingly, age, but not lameness significantly affected the time budget for locomotion and standing in this study, with old horses standing significantly more than younger horses. The standing time budget measured with the wearable tracking device in this study encompasses a broad repertoire of behaviors, including standing while eating or resting. The poor resolution of the standing behavior is one of the limitations of this study and would require the addition of other wearable equipment to remedy. Another limitation of this study is the low number of young and healthy horses. As the population of horses in animal shelters tend to include a large proportion of geriatric and lame horses, a more even distribution of groups was not possible within the framework of this study. To address this limitation, we also compared our results to time budgets reported for healthy adult horses in the literature. 

Recumbency times in this study were lower than the 203 +/− 46.5 min reported in the literature. However, the recumbency duration of adult horses has to date only been quantified in observational studies using scan or focal sampling, which may yield less detailed and accurate measurements or in polysomnographic analyses of equine sleep phases over 24 h only, which is insufficient time to measure cyclic recumbency patterns. The wearable automated tracking equipment used in this study enables the continuous measurement of recumbency, locomotion, and standing times over several days with a temporal resolution of one second. The ease of use, excellent tolerance of the sensor-bib by the horses, and automated app-based data analysis facilitate its use on-farm to identify horses with inadequate recumbency times or problems with locomotion, for welfare assessment and monitoring of the success of interventions. However, although the horses were housed in an animal sanctuary under appropriate management conditions, as is shown by the homogenous distribution of recumbency throughout the age and lameness groups, further optimization of environmental conditions, with individual adaptions to accommodate the unique population of horses living in an animal sanctuary, may increase recumbency times. 

## 5. Conclusions

Recumbency is a prerequisite for horses achieving rapid eye movement (REM) sleep and completing a full sleep cycle. Hence, measuring lying behavior is an essential component of equine welfare assessment. Wearable sensor technology can identify horses with low recumbency times and at risk for a REM-sleep deficit. Horses with REM deficit symptoms have not only lower recumbency but also decreased locomotion time budgets, indicating a general compromise of well-being. Interestingly, neither age nor lameness due to chronic orthopedic disease significantly influenced recumbency times in this study. Thus, geriatric horses and horses suffering from chronic orthopedic conditions can achieve recumbency times comparable to younger, healthy horses, but may require optimized husbandry conditions.

## Figures and Tables

**Table 1:** The recumbency duration is detailed by group (young lame, old lame, old sound, young sound) and by the presence of REM deficit symptoms. In addition, the number of horses per recumbency duration period is listed.

	*n*	Recumbency: Minutes/Day (Mean)	Recumbency: Minutes/Day (s.d.)	No Recumbency	Recumbency <10 min	Recumbency 10–30 min	Recumbency 30–60 min	Recumbency 60–120 min	Recumbency 120–180 min	Recumbency >180 min
**overall**	83	67.4	61.9	13	7	9	23	19	10	1
**young, lame**	31	85.0	70.3	7	1	2	6	9	5	0
**old, lame**	40	59.7	58.5	6	4	3	23	8	4	1
**old, sound**	7	36.5	26.5	0	1	3	2	1	0	
**young, sound**	5	64.1	50.7	0	1		2	1	1	
**REM deficit**	8	7.99	11.4	6	1	1	0			
**No REM deficit**	75	73.8	61.8	5	6	8	23	19	10	1

## Data Availability

All pertinent data is included in the manuscript and Supplementary Materials.

## Supplementary Materials

The following are available online at https://www.mdpi.com/article/10.3390/ani11113189/s1, Supplementary Table S1 details the age, sex, breed, group, the presence of lameness or REM deficit symptoms, BCS, access to pasture, and the time budgets (mean, s.d.) for lying, locomotion, and standing for all horses enrolled in this study. Supplementary Video S1: Horse collapsing due to REM-sleep deficit. Supplementary Video S2: Horse with REM sleep deficit falling asleep standing as it cannot lie down due to severe carpal osteoarthritis. Supplementary Table S2 details the recumbency time budgets per hour of the day for each horse enrolled in this study. Supplementary Figure S1: The distribution of recumbency times of three exemplary horses is shown for all 7–11 days they were tracked to demonstrate the strong individual variation. Horse A had long recumbency times and a regular daily rhythm, while horse B (the red line is not visible as it is continuously on the zero-line) never lay down (in 7 days), and horse C lay down only once in 11 days. Horses B and C showed REM deficit symptoms. Supplementary Table S3 details the locomotion time budgets per hour of the day for each horse enrolled in this study. Supplementary Table S4 details the standing time budgets per hour of the day for each horse enrolled in this study.
