# Supplementary figures and images for: Recumbency as an Equine Welfare Indicator in Geriatric Horses and Horses with Chronic Orthopaedic Disease
Source: Animals (Basel). 2021 Nov 8;11(11):3189. doi: 10.3390/ani11113189 (PMC8614510; doi:10.3390/ani11113189)

# Comparative Recumbency

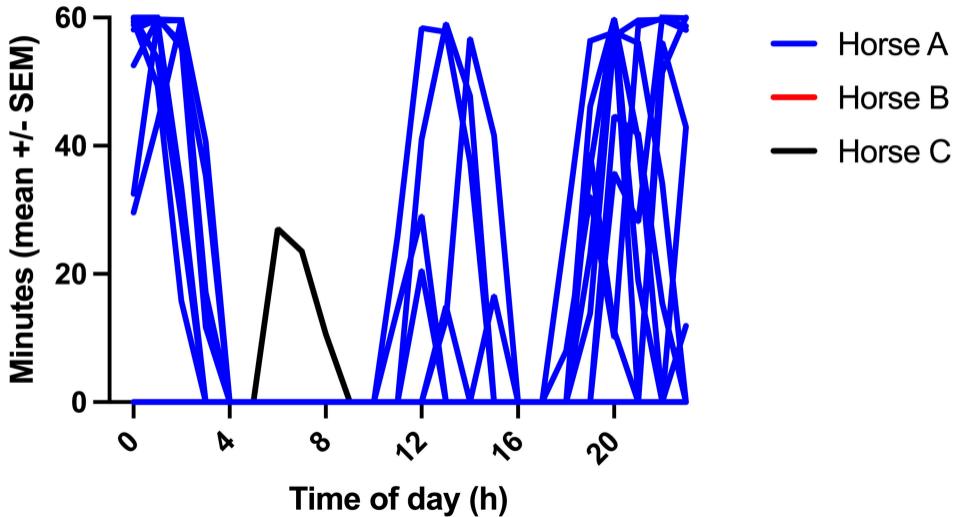

Supplement: Supplementary file 1 [file animals-11-03189-s001.zip › supplementary material/Suppl_fig_S5_example_distribution_lying .pdf]
